# Bone-marrow derived cells do not contribute to new beta-cells in the inflamed pancreas

**DOI:** 10.3389/fimmu.2023.1084056

**Published:** 2023-01-17

**Authors:** Yinan Jiang, John Wiersch, Wei Wu, Jieqi Qian, Maharana Prathap R. Adama, Nannan Wu, Weixia Yang, Congde Chen, Lingyan Zhu, Krishna Prasadan, George K. Gittes, Xiangwei Xiao

**Affiliations:** ^1^ Department of Surgery, Children’s Hospital of Pittsburgh, University of Pittsburgh School of Medicine, Pittsburgh, PA, United States; ^2^ Department of General Surgery, Children’s Hospital of Shanghai, Shanghai Jiao Tong University, Shanghai, China; ^3^ Department of Ultrasound in Medicine, the Second Affiliated Hospital Zhejiang University School of Medicine, Hangzhou, China; ^4^ Center for Endocrine Metabolism and Immune Diseases, Beijing Luhe Hospital, Capital Medical University, Beijing, China; ^5^ Department of Pediatrics, Affiliated Hospital of Nantong University, Nantong, China; ^6^ Department of Pediatric Surgery, the Second Affiliated Hospital and Yuying Children’s Hospital of Wenzhou Medical University, Wenzhou, China; ^7^ Department of Endocrinology, the First Affiliated Hospital of NanChang University, Nanchang, China

**Keywords:** acute pancreatitis, parabiosis, pancreatic duct ligation, pancreatic resident macrophages, bone marrow derived cells, inflammation

## Abstract

The contribution of bone-marrow derived cells (BMCs) to a newly formed beta-cell population in adults is controversial. Previous studies have only used models of bone marrow transplantation from sex-mismatched donors (or other models of genetic labeling) into recipient animals that had undergone irradiation. This approach suffers from the significant shortcoming of the off-target effects of irradiation. Partial pancreatic duct ligation (PDL) is a mouse model of acute pancreatitis with a modest increase in beta-cell number. However, the possibility that recruited BMCs in the inflamed pancreas may convert into beta-cells has not been examined. Here, we used an irradiation-free model to track the fate of the BMCs from the donor mice. A ROSA-mTmG red fluorescent mouse was surgically joined to an INS1^Cre^ knock-in mouse by parabiosis to establish a mixed circulation. PDL was then performed in the INS1^Cre^ mice 2 weeks after parabiosis, which was one week after establishment of the stable blood chimera. The contribution of red cells from ROSA-mTmG mice to beta-cells in INS1^Cre^ mouse was evaluated based on red fluorescence, while cell fusion was evaluated by the presence of green fluorescence in beta-cells. We did not detect any red or green insulin+ cells in the INS1^Cre^ mice, suggesting that there was no contribution of BMCs to the newly formed beta-cells, either by direct differentiation, or by cell fusion. Thus, the contribution of BMCs to beta-cells in the inflamed pancreas should be minimal, if any.

## Introduction

Postnatal beta-cell growth primarily results from beta-cell proliferation (1–3), while the contribution of stem/progenitor cells, especially bone-marrow derived cells (BMCs) including hematopoietic stem cells, mesenchymal stem cells, circulating endothelial progenitor cells and other cell types, to the newly formed pool of adult beta-cells is controversial (4–7). Previous studies have only used models of bone marrow transplantation from sex-mismatched donors (or other modes of genetic labeling) into recipient animals that had undergone irradiation (8). The fate of the grafted cells was then followed, taking advantage of either presence of Y chromosome or other tracible genetic labels from the donor cells (8). This approach suffers from the significant shortcoming of the off-target effects of irradiation that may affect not only the physiological homeostasis of the cells in the body, but also the microenvironment that controls the phenotype of the cells (9).

Partial pancreatic duct ligation (PDL) is a mouse model of acute pancreatitis, characterized by a robust infiltration of inflammatory cells into the ligated tail part of the pancreas. This model leads to a modest increase in beta-cell proliferation at early stages (10), and an epithelial-mesenchymal transdifferentiation-induced beta-cell loss in the long run (11), while the non-ligated head part of the pancreas remains largely unaffected (3, 12–14). The newly formed beta-cells in the inflamed tail of the pancreas have been suggested to partially derive from pancreatic duct cells (12), while other studies showed that the contribution of duct cells to newly formed beta-cells is small or none (15). Since many BMCs are recruited to the inflamed tail part of the pancreas (16), we thus wanted to assess whether those BMCs may contribute directly to newly-formed beta-cells.

Here, we used an irradiation-free parabiosis model to track the fate of the BMCs from the donor mice. Parabiosis is a surgical approach to generate a union of two organisms to develop a shared physiological circulating system. In this model, blood chimerism is formed from both animals to allow examination of the contribution of the circulating cells from one animal to cellular processes in the other (17). A ROSA-mTmG red fluorescent mouse was joined to an INS1^Cre^ knock-in mouse by parabiosis to establish a chimeric circulating. Red fluorescent white blood cells from ROSA-mTmG mice reached about 50% in both animals by 1 week after the surgery. PDL was then performed in the INS1^Cre^ mice 2 weeks after parabiosis surgery. The contribution of red cells from ROSA-mTmG mice to beta-cells in the INS1^Cre^ mouse was evaluated based on red fluorescence, while cell fusion was evaluated by presence of green fluorescence in beta-cells, which could occur when an existing beta-cell from the INS1^Cre^ mice fuses with a BMC-derived cell from the ROSA-mTmG mice. Our data suggest no contribution of BMCs to the newly formed beta-cells, either by direct differentiation or by cell fusion. Moreover, most inflammatory cells in the PDL-pancreas were macrophages derived from the circulation, so presumably not resident tissue macrophages.

## Methods

### Mouse manipulation

All mouse experiments were approved by the Animal Research and Care Committee at the Children’s Hospital of Pittsburgh and the University of Pittsburgh IACUC. C57/BL6, INS1^Cre^ knock-in (18) and ROSA-mTmG mice (19) were all purchased from the Jackson Lab (Bar Harbor, MA, USA). Since male mice are aggressive, only female mice were used in the current study for parabiosis. PDL was done as previously described (16). Briefly, after a midline incision was made in the upper abdominal quadrant, the mouse pancreatic main duct at pancreatic neck region was located under a stereoscopic microscope with the help of a sterile swab. Here the pancreatic duct was ligated with 6-G suture at the left side of the portal vein between the gastro-duodenal and splenic lobes. The inferior pancreaticoduodenal artery, superior pancreaticoduodenal artery and pancreatic part of the splenic artery should be minimally affected by the ligation. Parabiosis was done as previously described (17). Briefly, two mice were surgically joined first through their joints at elbow and knee with 4-G suture and then attached of their skin to allowing a firm connection without skin strain. Fasting blood sugar was measured as previously described (20).

### Pancreatic digestion and FACS

The digestion of the pancreas was performed as described previously (3). Briefly, pancreatic duct perfusion was performed, after which the pancreas was subsequently digested with 0.25 mg/ml collagenase (Sigma-Aldrich, Pittsburgh, PA, USA) for 30 minutes and with 10 μg/ml trypsin and 10 μg/ml DNase for approximately 20 minutes to obtain a single pancreatic cell population. Flow cytometry was done based on direct fluorescence for mT and CD45 or F4/80, using a pre-incubation with PE-cy7-conjugated anti-CD45 or anti-F4/80 (Becton-Dickinson Biosciences, San Jose, CA, USA), respectively.

### Immunohistochemistry

Immunohistochemistry for insulin (21) and CD45 (10) was done as previously described. Briefly, mouse pancreas underwent fixation in 4% formalin for 4 hours, followed by cryo-protection in 30% sucrose for 36 hours. The cryo-sectioning was performed at 6μm thickness of the tissue. Primary antibodies for immunostaining were rat polyclonal anti-CD45 (Becton-Dickinson Biosciences) and guinea pig anti-insulin (Dako, Carpinteria, CA, USA). Indirect fluorescent staining was obtained with either Cy2- or Cy3- conjugated secondary antibodies (Jackson ImmunoResearch Labs, West Grove, PA, USA). Nuclear staining was done with Hoechst 33342 solution (Invitrogen, Carlsbad, CA, USA).

### Data analysis

For *in vivo* experiments, 5 mouse pairs were used for each condition. Additional controls that were used for parabiosis ROSA-mTmG: INS1^Cre^ mice were INS1^Cre^: INS1^Cre^ mice and INS1^Cre^: INS1^Cre^mTmG mice. Quantifications were done in at least 6 slides with a distance of 100µm from each other per mouse pancreas or at least 5000 cells per sample. All data were statistically analyzed by one-way ANOVA with a Bonferroni correction. All error bars represent S.D. (standard deviation). Significance was presented as * when p<0.05. No significance was presented as NS. N values are either shown by individual data or by indicated in the figure legends.

## Results

### Establishment of an irradiation-free parabiosis model to track the fate of the BMCs in the PDL pancreas

**Figure 1:**
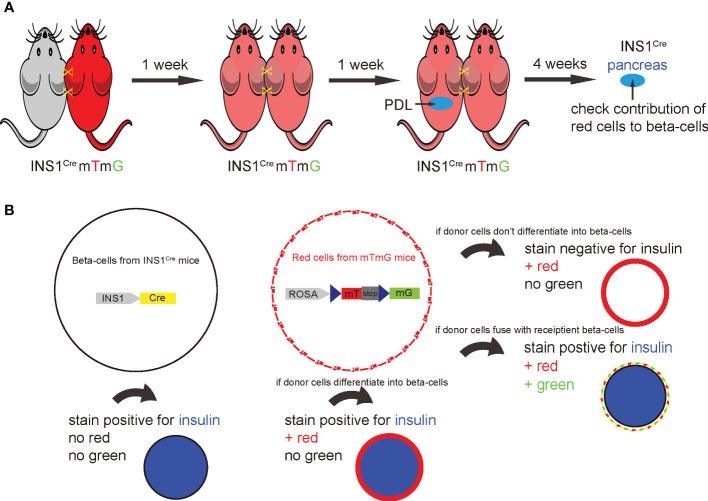
Establishment of an irradiation-free parabiosis model to track the fate of the BMCs in PDL pancreas. **(A)** Schematic to show parabiosis of a ROSA-mTmG red fluorescent mouse to an INS1^Cre^ knock-in mouse to establish a chimeric circulation. once the chimerism was established, PDL was performed on the INS1^Cre^ knock-in mice. Afterwards, the contribution of mT red cells derived from ROSA-mTmG mice to the beta-cell population in the PDL-INS1^Cre^ knock-in mice was assessed. **(B)** This system also allows detection of cell fusion, the occurrence of which could result in recombination of the loxp locus of ROSA-mTmG mice by the activated Cre recombinase in beta-cells from the INS1^Cre^ knock-in mice. The examination of insulin immunostaining along with detection of the mT red or mG green fluorescence of the cells together allows for the determination of the contribution of BMCs to beta-cells in the PDL-treated inflamed pancreas. The pre-existing beta-cells will stain positive for insulin, but negative for green mG or red mT fluorescence. If red cells from ROSA-mTmG mice do not stain positive for insulin, they do not directly contribute to beta-cells. If cells from ROSA-mTmG mice stain positive for insulin, but remain red and do not become green fluorescent, they directly contributed to beta-cells. If cells from ROSA-mTmG mice stain positive for insulin, but lose red fluorescent and become green, they must have fused with pre-existing beta-cells.

A ROSA-mTmG red fluorescent mouse was joined to an INS1^Cre^ knock-in mouse by parabiosis to establish a chimeric circulation. In this model, the red mT+ circulating cells from ROSA-mTmG mice gradually formed chimeric blood with the paired non-fluorescent INS1^Cre^ knock-in mice. After the chimeric circulation was established, PDL was performed on the INS1^Cre^ knock-in mice. Afterwards, the contribution of red cells derived from ROSA-mTmG mice to new beta-cells in the PDL-INS1^Cre^ knock-in mice was assessed (**Figure 1A**). This approach not only allowed for detection of direct conversion of circulation-derived cells into beta-cells, but also allowed for detection of cell fusion. In the event of cell fusion, the Cre recombinase from the beta-cells would activate the mG from the ROSA-mTmG locus. Thus, overall, insulin immunostaining and analysis of direct fluorescence (mT or mG) would determine potential contribution of BMCs to beta-cells in the PDL-treated inflamed pancreas (**Figure 1B**). The pre-existing beta-cells will stain positive for insulin, but negative for green mG or red mT fluorescence. If red cells from ROSA-mTmG mice do not stain positive for insulin, they did not directly contribute to beta-cells, but were merely present in the pancreas. If cells from the ROSA-mTmG mice stain positive for insulin, but remain red and do not become green fluorescent, they directly contributed to beta-cells and are not green because they do not have cre recombinase. If cells from ROSA-mTmG mice stain positive for insulin, but lose their red fluorescent and become green, they must have fused with pre-existing beta-cells (**Figure 1B**).

### A stable chimeric circulation is established by 7 days after parabiosis surgery

**Figure 2:**
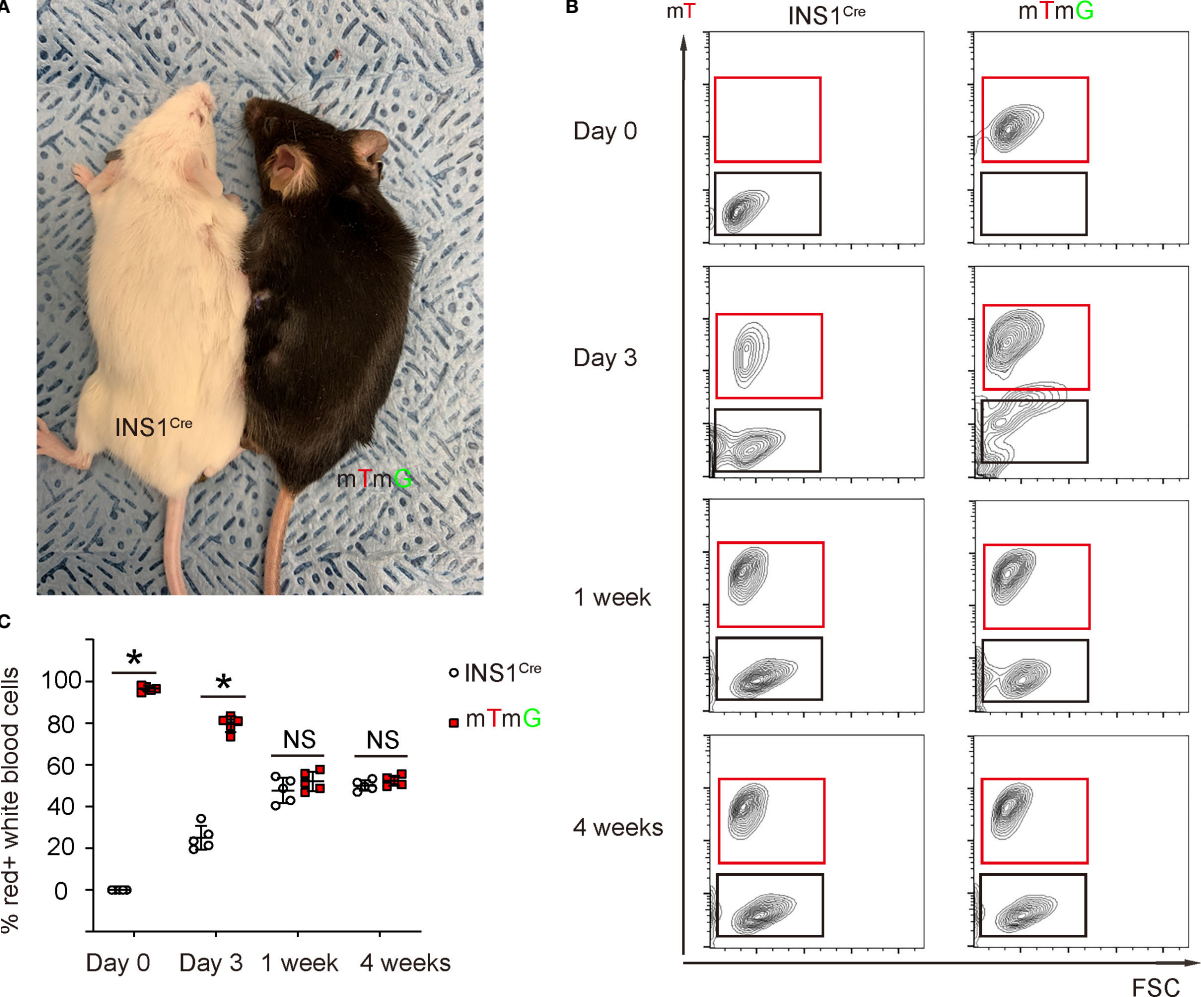
Stable blood chimerism is established as early as 7 days after parabiosis. **(A)** A gross image showing a ROSA-mTmG mouse and PDL-INS1^Cre^ knock-in mouse after parabiosis. **(B, C)** Blood was taken from both mice at serial time points after parabiosis to analyze the degree of chimerism. mT was analyzed by flow cytometry, shown by representative flow charts **(B)** and by quantification **(C)**. *p <0.05. NS: non-significant. N=5 repeats.

We analyzed the length of time required to generate a stable chimeric circulation in mice after parabiosis surgery (**Figure 2A**). For this analysis, blood was taken from each mouse of the pair at serial time points to determine the degree of chimerism. We did not detect red cells in INS1^Cre^ knock-in mice before parabiosis. Moreover, as early as day 3 after parabiosis, significant blood exchange was already detected in both animals (**Figures 2B, C**). At day 7 after parabiosis, the chimerism rate reached about 50% and became stable (**Figures 2B, C**). Thus, stable blood chimera is established within 7 days after parabiosis surgery. To ensure that the PDL was performed at a time when the circulation was fully chimeric, we performed the PDL 2 weeks after parabiosis.

### PDL does not alter blood glucose in either mouse after parabiosis

**Figure 3:**
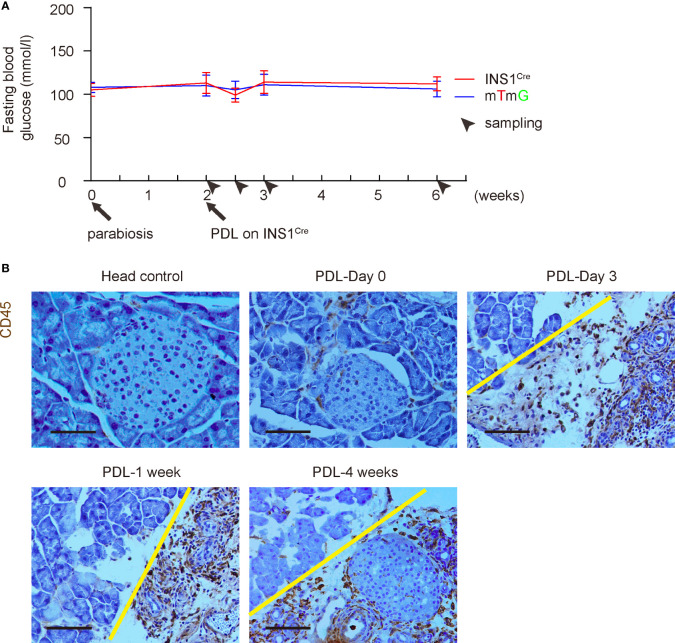
PDL does not alter blood glucose in either mouse after parabiosis. **(A)** Fasting blood glucose levels in both mice after parabiosis/PDL. **(B)** Representative immunohistochemical images for CD45 staining. Head control, a control of non-ligated PDL-head from the INS1^Cre^ knock-in mouse (PDL 1week). Others are from the ligated PDL-tail from the INS1^Cre^ knock-in mouse at different time points. Yellow line separated head (left) from tail (right) part of the pancreas. Scale bars are 100 µm.

We and others have previously shown that PDL does not alter blood glucose levels since half of the pancreas (head part of the pancreas) remains unaffected (3, 12–14). Here, we also followed the blood glucose levels up to 4 weeks after PDL. We did not detect alterations in blood glucose levels, suggesting that parabiosis itself does not have effects on the glycemia of PDL-mice (**Figure 3A**). CD45 immunostaining was performed on the INS1^Cre^ knock-in mice at different time points after PDL, showing similar inflammatory infiltration and histological alterations in these mice (**Figure 3B**), compared to PDL-pancreas without parabiosis (10, 16).

### mT red inflammatory cells are detected in the inflamed PDL pancreas

**Figure 4:**
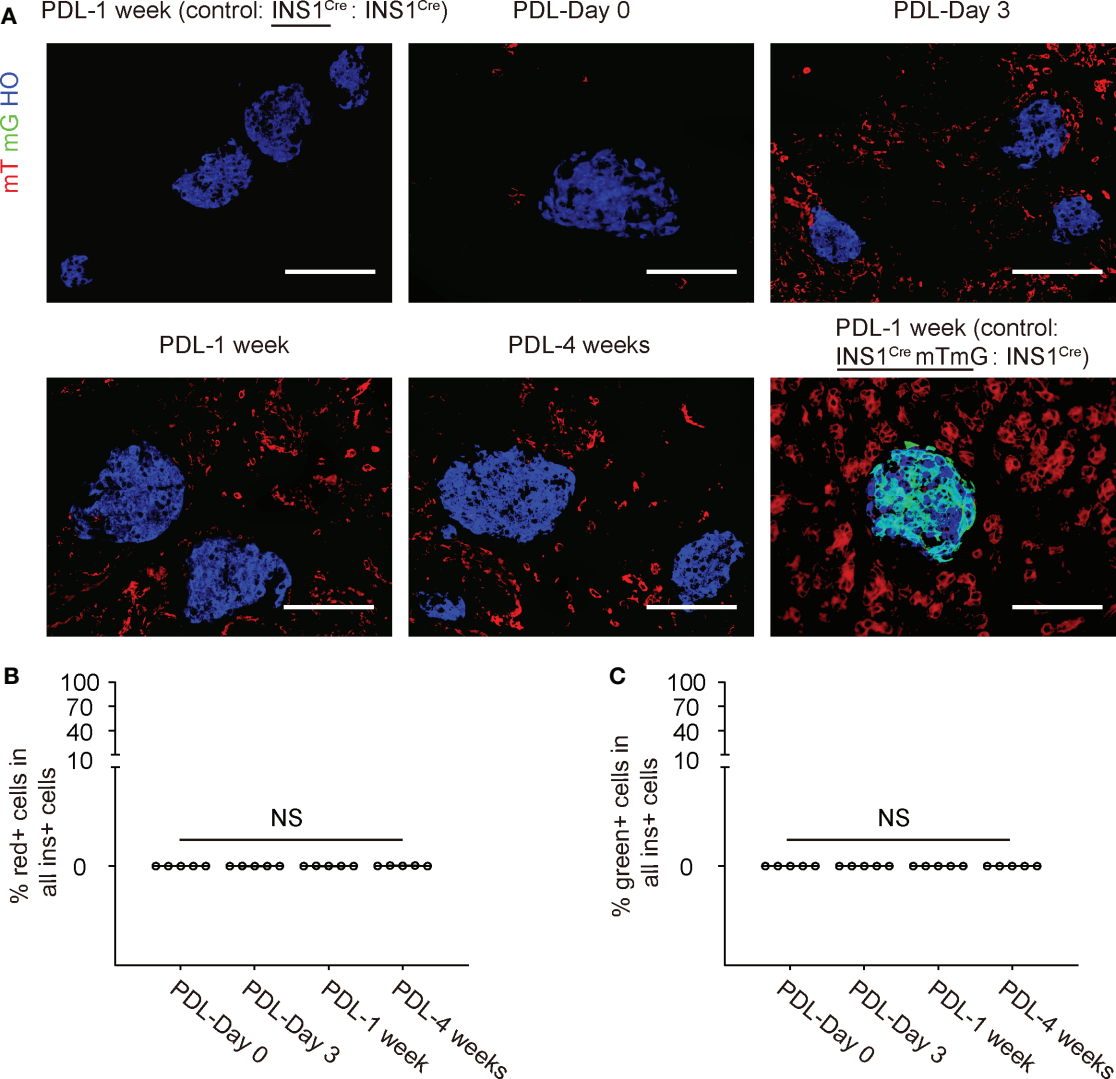
No red/green beta-cells are detected in the inflamed PDL pancreas. **(A)** Representative immunohistochemical images for mT, mG and insulin. Besides the ligated PDL-tail from the INS1^Cre^ knock-in mouse at different time points, two controls were shown. The first control was the PDL-tail from a control parabiosis by joining two INS1^Cre^ knock-in mice together, and one of the mice received PDL at the same time point as the experimental groups. This was the negative control for red fluorescence. The other control was the PDL-tail from another control parabiosis by joining an INS1^Cre^mTmG mouse with an INS1^Cre^ knock-in mice together, and the INS1^Cre^mTmG mouse received PDL at the same time point as the experimental groups. This was the positive control for green fluorescence to detect cell fusion. **(B)** Quantification of the mT red **(B)** or mG green **(C)** beta-cells in the inflamed pancreas at different time points after PDL in INS1^Cre^ knock-in mice that were joined to the ROSA-mTmG mice by parabiosis. NS: non-significant. N=5 repeats. Scale bars are 100 µm.

No mT red fluorescent cells were detected in the control INS1^Cre^ knock-in mice that were joined with another INS1^Cre^ knock-in mice in a parabiosis either before or after PDL (image 1 week after PDL is shown in **Figure 4A**). Only rare mT red fluorescent cells were detected before PDL in INS1^Cre^ knock-in mice that were joined to the ROSA-mTmG mice by parabiosis. However, many mT red cells were detected in the PDL-pancreas starting from 3 days after PDL (**Figure 4A**).

### No red/green beta-cells are detected in the inflamed PDL pancreas

We looked for mT red (**Figure 4B**) or mG (**Figure 4C**) beta-cells in the inflamed pancreas at different time points after PDL in INS1^Cre^ knock-in mice that were joined to the ROSA-mTmG mice by parabiosis, but we did not detect any. These data suggest that there is no direct contribution of BMCs to beta-cells in the PDL-pancreas.

### Inflammatory cells in the PDL pancreas are mainly recruited from the circulation

**Figure 5:**
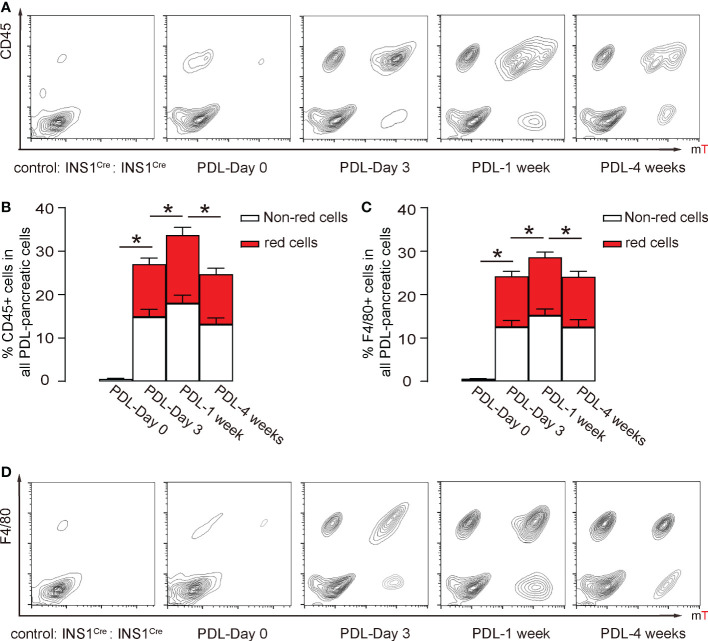
Inflammatory cells in the PDL pancreas are mainly recruited from the circulation. **(A, B)** Flow cytometry analysis of red fluorescent and CD45+ cells in the digested pancreas, shown by representative flow charts **(A)** and by quantification of (red) CD45+ cells in all PDL-pancreatic cells **(B)**. **(C, D)** Flow cytometry analysis of red fluorescent and F4/80+ cells in the digested pancreas, shown by quantification of (red) F4/80+ cells in all PDL-pancreatic cells **(C)** and by representative flow charts **(D)**. *p<0.05. N=5 repeats.

Pancreatic macrophages have two origins, one is residential macrophages, and the other is circulation-derived macrophages. This parabiosis model allows examination of the contribution of these two populations to pancreatic inflammation. We assessed the pancreatic inflammatory cells by flow cytometry. First, the digested PDL-pancreas was analyzed at different time points for a pan-leukocyte marker CD45 and red fluorescence. Only rare CD45+ inflammatory cells were detected before PDL, but then CD45+ inflammatory cell numbers dramatically increased as early as day 3 after PDL, peaked at 1 week after PDL, and went down at 4 weeks after PDL (**Figures 5A, B**). Moreover, red CD45+ cells were just slightly less than half of total CD45+ cells (**Figures 5A, B**). Since the circulation is 50% derived from each mouse, these data suggest that most of the CD45+ cells in the inflamed pancreas were recruited from circulation, consistent with a previous study (22). Similar results were obtained using a macrophage-specific marker F4/80 instead of CD45 (**Figures 5C, D**), while the total percentage of F4/80+ cells was slightly lower than CD45+ cells (**Figures 5B, C**). These data suggest that the majority of inflammatory cells in the PDL-pancreas were recruited macrophages from the circulation.

## Discussion

Transplantation of BMCs offers the advantage of autologous transplantation, which has been claimed by some to promote (partial) recovery from hyperglycemia (4, 5). In contrast, others did not report beneficial effects of transplantation of BMCs in diabetic mice (6, 7, 23, 24). Moreover, the mechanism by which these cells potentially support beta-cell neogenesis and regeneration has been poorly addressed and various inconsistent hypotheses have been proposed. Some groups reported that adult BMCs can differentiate at variable efficiency into insulin-expressing cells when injected into irradiated normoglycemic (25) or diabetic mice (5, 23). On the other hand, it has been documented that some of these cells express endothelial markers following transplantation in diabetic mice, resulting in no (6, 7, 23, 24) or only marginal (5) improvement in hyperglycemia. Others reported the differentiation of engrafted BMCs towards CD45^+^ cells (4, 24). Of note, none of these studies have used a radiation-free method to avoid off-target effects, and the examined models did not involve a severe pancreatic inflammation. Here, our approach addressed both of these issues. First, parabiosis is a model that allows a gradual formation of blood and bone marrow chimera without the need for irradiation, thus avoiding off-target effects to the maximum. Second, PDL is a model with an increase in beta-cell numbers over a relatively short period (7-30 days) (3, 12), and it is an inflammatory model mainly involving BMC-derived immune cells (16, 26).

Interestingly, we found a large number of mT red cells infiltrated in the inflamed pancreas after PDL, starting as early as day 3. Most of those cells were also found to be CD45+, mainly consisting of F4/80+ macrophages. Some CD45- red cells could be derived from mesenchymal stem cells or circulating endothelial precursor cells, which were known to express little CD45 (27). All red cells were certainly derived from the chimeric circulation, not from the local pancreatic resident cells, since the resident cells should be non-fluorescent. Past studies have shown a diverse role of resident macrophages versus circulating monocytes/macrophages in different organs and under different situations (28). Moreover, we and others have shown the dynamic changes in macrophage M1/M2 phenotypes after PDL (10, 22). Since PDL is a harsh and acute insult to the pancreas, it is understandable that the resident macrophages do not have time to respond adequately and thus the circulating cells are committed to assist this urgent request from the pancreas and participate in the response. On the other hand, pancreatic resident macrophages have been shown to play a more important role in other circumstances like obesity (29). It may be interesting to determine the exact molecular signaling that controls the resident macrophages and circulating monocytes/macrophages under different circumstances in a future study. Moreover, the normoglycemia in PDL model does not allow examination of BMC-to-beta-cell conversion under a hyperglycemic condition, which is a limitation of the current study and could be addressed later in an approach that applies toxin-induced diabetes into the current model.

Given the lack of radiation in this model that involves an extensive presence of BMCs, our finding of little or no contribution of BMCs to beta-cells creates a strong argument against the possibility of significant differentiation of BMCs into insulin-producing beta-cells in the adult.

## Data availability statement

The original contributions presented in the study are included in the article/supplementary material. Further inquiries can be directed to the corresponding authors.

## Ethics statement

The animal study was reviewed and approved by University of Pittsburgh.

## Author contributions

YJ, JW, WW, JQ, MA, NW, WY, CC, LZ, KP, GG and XX were responsible for data acquisition and analysis. XX wrote the manuscript. XX and GG were responsible for study conception, design and funding and are the guarantors of the study. All authors contributed to the article and approved the submitted version.
